# Are We Ready for a True Biopsychosocial–Spiritual Model? The Many Meanings of “Spiritual”

**DOI:** 10.3390/medicines4040079

**Published:** 2017-10-31

**Authors:** Marcelo Saad, Roberta de Medeiros, Amanda Cristina Mosini

**Affiliations:** 1 Spiritist Medical Association of São Paulo, Av. Pedro Severino Junior, 323, Jabaquara, São Paulo 04310-060, Brazil; 2 Department of Physiology, Centro Universitário São Camilo, São Paulo 04263-200, Brazil; robertademedeiros@uol.com.br; 3 Department of Physiology, Universidade Federal de São Paulo, São Paulo 04021-001, Brazil; amandacfmosini@gmail.com

**Keywords:** humanities, medical philosophy, spirituality, holistic health care, integrative medicine, soul–body relations

## Abstract

The biopsychosocial model is a modern humanistic and holistic view of the human being in health sciences. Currently, many researchers think the biopsychosocial model should be expanded to include the spiritual dimension as well. However, “spiritual” is an open and fluid concept, and it can refer to many different things. This paper intends to explore the spiritual dimension in all its meanings: the spirituality-and-health relationship; spiritual–religious coping; the spirituality of the physician affecting his/her practice; spiritual support for inpatients; spiritual complementary therapies; and spiritual anomalous phenomena. In order to ascertain whether physicians would be willing to embrace them all in practice, each phrase from the Physician’s Pledge on the Declaration of Geneva (World Medical Association) was “translated” in this paper to its spiritual equivalent. Medical practice involves a continuous process of revisions of applied concepts, but a true paradigm shift will occur only when the human spiritual dimension is fully understood and incorporated into health care. Then, one will be able to cut stereotypes and use the term “biopsychosocial–spiritual model” correctly. A sincere and profound application of this new view of the human being would bring remarkable transformations to the concepts of health, disease, treatments, and cure.

## 1. The Biopsychosocial Model and the Spiritual Dimension

The biopsychosocial model is a modern humanistic and holistic view of the human being. The model was brought to medicine by George L. Engel (1913–1999), a prominent scholar engaged in the psychosomatic movement [1]. As Engel stated: “all three levels, biological, psychological, and social, must be taken into account in every health care task” [2]. His claim was that physicians must simultaneously attend to the biological, psychological, and social dimensions of illness, in order to better understand and respond to patients’ suffering. A perfect description of the biopsychosocial model was given by Borrell-Carrió et al. [3]: “It is both a philosophy of clinical care and a practical clinical guide. Philosophically, it is a way of understanding how suffering, disease, and illness are affected by multiple levels of organization, from the societal to the molecular. At the practical level, it is a way of understanding the patient’s subjective experience as an essential contributor to accurate diagnosis, health outcomes, and humane care”. In the last decades, the humanization of medicine and empowerment of patients have been constantly improved by including the patient’s subjective experience, by expanding the disease causational framework to a more comprehensive model, by valuating the patient–clinician relationship, and by giving new roles to the patient in clinical decision-making.

Currently, many researchers think the biopsychosocial model should be expanded to include the spiritual dimension as well. One such researcher is Katerndahl [4], whose study has shown the relevance of spiritual symptoms and their interactions for understanding health outcomes. Sulmasy [5] justifies the expansion of the model to a biopsychosocial–spiritual one by remembering that genuinely holistic health care must address the totality of the patient’s relational existence. According to him, this will contribute to a more comprehensive model of care and research that takes account of patients in their fullest wholeness. Arguably, the transcendent and sacred questionings of the spiritual dimension cannot be exhausted on the mental and social grounds, notwithstanding the interfaces between the concepts.

“*Health is a state of complete physical, mental and social well-being, and not merely the absence of disease or infirmity*.” In this definition, adopted in 1948 by the World Health Organization (WHO) after its foundation, the spiritual dimension was absent. However, in 1999, the 52nd Assembly of this institution proposed some amendments to its Constitution. One of the proposed modifications was the insertion of spiritual well-being into the WHO concept of health [6]. The new text would become “*Health is a dynamic state of complete physical, mental, spiritual and social well-being and not merely the absence of disease or infirmity*”. Even so, despite approval during the Assembly, the new version was posteriorly vetoed. Notwithstanding, in many occasions on the last decades, the WHO highlighted the importance of the spiritual dimension for clinical purposes.

## 2. The Many Meanings for “Spiritual”

“Spiritual” is today an open and fluid concept. It can refer to many different aspects, from non-religious and non-theistic levels (such as the power of positive thinking) to deeply religious experiences [7]. The contemporary pluralistic society and individualistic culture allow different people to have diverse interpretations about the term “spiritual”. This paper intends to explore the spiritual dimension in all its meanings. Below, the reader will find descriptions for some meanings to the word “spiritual” related to health purposes.

Spirituality-and-Health Relationship: In the health sciences, spirituality has received many definitions by different authors. Perhaps the most comprehensive definition poses spirituality as “the search for ultimate meaning, purpose and significance, in relation to oneself, family, others, community, nature, and the sacred, expressed through beliefs, values, traditions and practices” [8]. Many people express their spirituality on their formal religions or their traditional faiths. Others still strengthen their spiritual dimension with non-religious elements. Although religiosity and spirituality are distinct constructs, the overlap between them is remarkable and consistent. Thus, the term spiritual–religious (S-R) is often adopted to refer to transcendent elements and connectedness to life essence. There is extensive scientific documentation about the positive beneficial association between the binomial S-R and clinical parameters of physical and mental health, culminating in increased quality of life and longevity [9]. This effect is not only statistically significant but also clinically relevant.

Spiritual–Religious Coping: This refers to ways that individuals utilize a personal S-R framework to reduce the emotional distress caused by adverse events of life, such as loss or change. S-R beliefs, attitudes, or practices may give meaning for suffering, thus making it more bearable. Such approach could regulate stress during circumstances that are out of patients’ personal control. This positive S-R coping is a valuable resource of strength and hope, which should be encouraged by the physician [10]. Some faith-based techniques for health purposes, such as repetitive prayer, can indeed produce stress relief [11]. On the other hand, S-R coping may sometimes be harmful, when it is based on negative feelings such as anger, sorrow, guilt or stigma. Such negative S-R coping produces spiritual struggle that must be identified and addressed; otherwise it will adversely affect the course of disease, by worsening stress.

Spirituality of the Physician Affecting His/Her Practice: The S-R characteristics of physicians may have great relevance to the professional practice. The physician’s religious commitments or moral perspectives may direct clinical decisions, especially on sensitive issues such as interruption of pregnancy or treatment suspension in terminal illnesses [12]. In addition, the importance given by the physician to the spirituality of the patient during the clinical encounter may be related to the physician’s own spirituality [13]. The S-R characteristics of the physician can also be a resource for facing the stress and challenges of the profession, not only preventing burnout but also improving job satisfaction [14]. Such characteristics could also influence the view of the profession as a ministry, a sacred calling to help others, almost like a higher vocation [15]. According to Cole [16], humanizing medicine depends on recovering the humanity of physicians. The author proposed actions for academic institutions in order to humanize the experience of doctoring, such as the provision of programs that support and encourage self-care, personal reflection, and lifestyle choices.

Spiritual Support for Inpatients: A contemporary orientation of the hospital experience model must encompass the spiritual dimension. In order not to hurt sensibilities or be invasive, the ideal situation is to check with the patient, on admission, whether he/she wants an S-R visit. If so, the patient name goes into a list that is provided to the clergyman, who then makes the religious visit only for them, avoiding an inopportune intrusion. In a routine screening, desirable items include the record of the belief system (affiliation); level of religious observance; involvement with religious community; and particularly important rituals [17]. In health care institutions, chaplains typically serve people of many different denominations, in a multifaith effort. If the hospital does not have a chaplain, external clergymen may make occasional visits. Alternatively, for less specialized needs, laic volunteers may support universal questions without discussing beliefs, through presence, compassion, and understanding. The minimum role of the physician, as an important agent in this process, is to identify proactively S-R needs and to trigger the available supportive resources.

Spiritual Complementary Therapies: Spiritual therapies (STs) may be defined as “mystical, religious, or spiritual practices performed for health benefit” [18]. This term encompasses a broad group of modalities, including millenarian- or contemporary-created ones, the religious- or secular-oriented ones, and the professionally- or empirically-learned ones. Some STs involve the practitioner touching the patient, laying their hands on them, or simply directing his/her mental intention at distance. Examples of STs include Healing Touch, Johrei, Reiki, Therapeutic Touch, and blessings from many religious traditions. Based on available scientific evidence, the supposed “vital energy”, implicated with the process is just conjecture. Besides, the clinical utility of ST is limited due to the lack of uniformity of responses and the low clinical relevance from a small decrease in symptoms. Even so, the health care professionals must construct a line of reasoning about ST, since their patients may ask for advice about them. A humanistic, comprehensive and integrative health care must include such complementary approaches without obstacles. The role of the physician is to accompany the patient in his/her pursuit, remaining sympathetic and respectful to their aspirations and desires. Complementary therapies come at low or no cost, can produce a useful placebo response, and are seldom harmful unless relied on as an alternative to professional advice and treatment.

Spiritual Anomalous Phenomena: For a long time, many studies with correct methodology have documented disconcerting events and experiences related to anomalous phenomena of consciousness [19]. Unexplained events related to mystical experiences are regularly reported in the medical literature [20]. Some scholars anticipate the need for a new scientific paradigm that considers the existence of non-physical levels of reality [21]. There is an increasing amount of research exploring the idea that the mind is a separate entity to the brain, an assumption called “possibility of consciousness independent from the body” [22]. The physician may face unexplained events such as a near-death experience or memories of a supposed past life. Embracing the patient’s spiritual belief will broaden our understanding of human nature, enhance our cultural sensitivity, and improve the effectiveness of mental health initiatives [23]. Psychotherapeutic approaches should respect patient subjective realities, such as belief in life after death, as a therapeutic need and an ethical duty, even though therapists may not share the same belief [24]. Approaches like past-life regressive therapy, by restructuring a trauma in a supposed past life, could successfully treat problematic personal relationships, phobias, and a lack of meaning and purpose in life [25].

## 3. Are We Ready?

In order to ascertain whether physicians would be willing to embrace them all in practice, Table 1 poses a challenge. It brings all the phrases from Physician’s Pledge on the Declaration of Geneva. Each one confronted with its “translation” to the spiritual dimension. The Declaration of Geneva was intended as a revision of the Hippocratic Oath’s moral values to a formulation that could be comprehended and acknowledged in a modern way. This pledge has been adopted by the World Medical Association (WMA) in 1948 as a declaration of a dedication to the humanitarian goals of medicine. Through the decades, the text has been modified several times, with the most recent amendment made on October 2017 during the 68th WMA General Assembly in Chicago (USA) [26].

## 4. Conclusions

Medical practice involves a continuous process of revision of applied concepts. In the last decades, the health care experience has evolved from disease-centered care (hospital as a “biological garage”) to patient-centered care (humanization movement) and, more recently, to person-centered care (personalization movement). In this process, the spiritual dimension gained progressively more relevance. Currently, spirituality has paramount importance for high standard medical training and clinical practice. This is notably associated to mental health, as recently reported by the World Psychiatric Association [27]. However, a true paradigm shift will occur only when the human spiritual dimension is fully understood and incorporated in health care. Then, one will be able to cut stereotypes and properly use the term “biopsychosocial–spiritual model”. A sincere and profound application of this new view of the human being would bring remarkable transformations to the concepts of health, disease, treatment, and cure.

## Figures and Tables

**Table 1:** Phrases from Physician’s Pledge on the Declaration of Geneva, each one confronted with its “translation” to the spiritual dimension.

Phrases from the Physician’s Pledge	“Translation” to the Spiritual Dimension
As a member of the medical profession, I solemnly pledge to dedicate my life to the service of humanity;	I see medicine as a spiritual calling for the higher good of others, almost like a sacred fellowship (we are all one)
The health and well-being of my patient will be my first consideration;	I will value the spiritual values, beliefs and practices of my patient in the clinical encounter, with respect their impact on his/her health
I will respect the autonomy and dignity of my patient;	I shall not impose my spiritual beliefs on any patient
I will maintain the utmost respect for human life;	If abortion and euthanasia are legal in my country, I will invoke universal spiritual values regarding life as sacred to frame my clinical conduct
I will not permit considerations of age, disease or disability, creed, ethnic origin, gender, nationality, political affiliation, race, sexual orientation, social standing or any other factor to intervene between my duty and my patient;	The multidimensional diversity of patients is an invitation to accommodate my clinical practice sympathetically in light of their deeply personal spiritual–religious experiences, values, beliefs and practices
I will respect the secrets that are confided in me, even after the patient has died;	When a patient brings to me spiritual concerns, I will embrace them as an essential aspect of the clinical planning
I will practice my profession with conscience and dignity and in accordance with good medical practice;	I consider my ethical commitments and community engagement as laic manifestations of my personal spirituality, reflecting universal values and truths about humanity.
I will foster the honour and noble traditions of the medical profession;	I will remember that, throughout most of human history, healing practice was linked to spiritual guidance
I will give to my teachers, colleagues, and students the respect and gratitude that is their due;	By way of gratitude and respect, I will hold these people in my prayers (or I will ever commit my thoughts to their well-being)
I will share my medical knowledge for the benefit of the patient and the advancement of health care;	I will develop my medical knowledge and expertise by taking into account all good-quality scientific reports on spirituality and health care, including studies of spiritually anomalous phenomena
I will attend to my own health, well-being, and abilities in order to provide care of the highest standard;	I will remember that spirituality is important for bringing me comfort and strength, improving my capacity for both clinical and inter-personal discernment, and helping me cope with professional stress
I will not use my medical knowledge to violate human rights and civil liberties, even under threat;	I shall engage in spiritual practices to strengthen my spiritual values, growing in compassion and wisdom as part of the key internal guides to my moral behavior
I make these promises solemnly, freely and upon my honour.	May I grow spiritually through the relationship with my patients, their families and my colleagues through the privilege of practicing medicine
